# A Versatile Toolset for Genetic Manipulation of the Wine Yeast *Hanseniaspora uvarum*

**DOI:** 10.3390/ijms24031859

**Published:** 2023-01-17

**Authors:** Jürgen J. Heinisch, Andrea Murra, Kai Jürgens, Hans-Peter Schmitz

**Affiliations:** 1 AG Genetik, Fachbereich Biologie/Chemie, Universität Osnabrück, Barbarastr. 11, D-49076 Osnabrück, Germany; 2 AG Zoologie, Fachbereich Biologie/Chemie, Universität Osnabrück, Barbarastr. 11, D-49076 Osnabrück, Germany

**Keywords:** wine yeast, transformation, dominant selection markers, auxotrophic markers, Cre/loxP system

## Abstract

*Hanseniaspora uvarum* is an ascomycetous yeast that frequently dominates the population in the first two days of wine fermentations. It contributes to the production of many beneficial as well as detrimental aroma compounds. While the genome sequence of the diploid type strain DSM 2768 has been largely elucidated, transformation by electroporation was only recently achieved. We here provide an elaborate toolset for the genetic manipulation of this yeast. A chromosomal replication origin was isolated and used for the construction of episomal, self-replicating cloning vectors. Moreover, homozygous auxotrophic deletion markers (*Huura3*, *Huhis3*, *Huleu2*, *Huade2*) have been obtained in the diploid genome as future recipients and a proof of principle for the application of PCR-based one-step gene deletion strategies. Besides a hygromycin resistance cassette, a kanamycin resistance gene was established as a dominant marker for selection on G418. Recyclable deletion cassettes flanked by *loxP*-sites and the corresponding Cre-recombinase expression vectors were tailored. Moreover, we report on a chemical transformation procedure with the use of freeze-competent cells. Together, these techniques and constructs pave the way for efficient and targeted manipulations of *H. uvarum*.

## 1. Introduction

The yeast *Saccharomyces cerevisiae* is undoubtedly the first and most frequently employed biotechnological production organism used by mankind for millennia in the manufacture of bread and alcoholic beverages [1]. However, the quality of fermented products is invariably determined by its co-existence with other yeast and bacterial species and their metabolic activities [2,3,4]. Especially in the production of wine, beer, and cider, *Hanseniaspora uvarum* is often predominating in the early stages of spontaneous fermentations, worldwide, as it is readily found on the surface of grapes and apples, but also on various other fruits, such as plums and strawberries [5,6,7,8]. Even in coffee and chocolate bean fermentations, *H. uvarum* contributes significantly to the final product quality [9,10]. In addition, *H. uvarum* and other endophytic yeasts have been shown to promote growth of their host agricultural fruit plants by producing phytohormones [5]. Moreover, its potential as a biological control agent to diminish the loss of fruit harvests by competing with other yeasts and fungi is increasingly being recognized [6,11,12,13,14]. *Hanseniaspora uvarum* can also be employed in another type of biological control to fight *Drosophila suzukii*, which already causes fruit harvest losses in Southern Europe, especially of grapes and cherries [15]. The strong attraction of the fruit flies to *H. uvarum*, presumably due to the efficient production of ester compounds like ethyl acetate, is thus employed to build traps for efficient pest management [16,17]. Interestingly, but not yet applied in oenology or medicine, *H. uvarum* also produces a killer toxin with activity against *Candida* species [18].

Despite these emerging applications, the biology of *H. uvarum* has been most extensively studied for its contributions in wine making, as in the initial stages of must fermentations it may not only prevail under spontaneous conditions, but also upon addition of *S. cerevisiae* starter cultures [19,20]. While, in experimental co-fermentations with other yeasts, *H. uvarum* can improve the richness in taste by contributing a variety of positive aroma compounds, especially fruity esters [21,22,23,24,25], it may also produce unwanted high amounts of acetic acid, as well as exceedingly high levels of ethyl acetate, depending on the inoculation conditions [26]. Such co-fermentations have also been investigated for their capacity to reduce the final ethanol content [27,28] to combat problems induced by climate-change and social awareness. The limited capacity for alcoholic fermentation and production of high levels of acetic acid may vary between different isolates of *H. uvarum* [29]. The yeast is classified as Crabtree-negative, meaning that it consumes glucose mainly through respiration even at high sugar concentrations. It has been studied in chemostat cultures, attributing the production of acetate to a limited activity of acetyl-CoA synthetase [30]. Later, determinations of glycolytic enzyme activities in comparison to those of *S. cerevisiae* indicated that pyruvate kinase could be the limiting step for alcoholic fermentation in *H. uvarum* [31]. Apart from such intrinsic metabolic capacities, yeasts may also contribute to wine quality by the export of cell wall degrading enzymes, like ß-glucanases, through extracellular vesicles [32].

Regarding the genetics of *H. uvarum*, few studies have been published and knowledge remains fairly scarce [33]. The genome sequence has been determined for a few different isolates of this species [31,34,35], with a considerable annotation and a draft genomic organization of *H. uvarum* DSM 2768 (also designated as CBS 2584 or NRRL 115) provided in [31]. A recent large-scale genomic analysis dedicated to the analysis of hybridization between different species of *Hanseniaspora* revealed only one triploid isolate of *H. uvarum*, but no interspecies hybrid [36]. No reports on targeted genetic manipulation were available until the break-through work of Badura et al. [37], which developed a transformation procedure based on electroporation and a dominant selection marker conferring resistance to hygromycin B. This marker was used a second time by increasing the concentration of the antibiotic in a strain in which the first copy of the *HuATF1* gene had already been deleted, to remove both copies from the diploid genome of strain *H. uvarum* DSM 2768. Nevertheless, the lack of a second selection marker and of autonomously replicating plasmids made this procedure time-consuming and difficult to reproduce in large-scale manipulations.

We, therefore, here developed a set of episomal vectors, auxotrophic mutants, and re-usable marker cassettes for efficient deletions of both alleles of genes of interest from the *H. uvarum* genome. Moreover, a chemical transformation procedure based on freeze-competent cells greatly facilitates the handling of this yeast in genetic manipulations.

## 2. Results

### 2.1. Characterization of the HHO1 Recipient Strain

**Figure 1:**
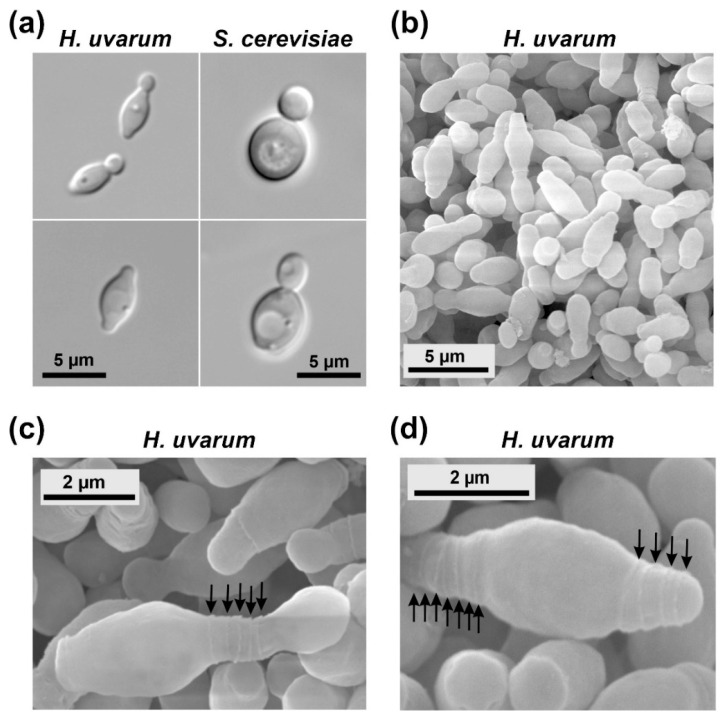
Images of budding yeast cells. (**a**) Differential interference contrast images of logarithmically growing cells of *Hanseniaspora uvarum* strain HHO1 (left panel) and *Saccharomyces cerevisiae* DHD5 (right panel). The upper and lower panels show cells in different stages to demonstrate the typical differences in the budding pattern of the two yeast species. The upper image for *H. uvarum* shows cells believed to be at the end of cytokinesis, whereas the lower image represents a cell after budding, presumably in the G1 phase of the cell cycle. Size bars are indicated and applicable to both images of the same yeast. (**b**–**d**) Images from scanning electron microscopy (SEM) of a logarithmically growing culture of *H. uvarum* HHO1, showing single-celled growth by bipolar budding, generating the typical lemon-shaped cells. Size bars are indicated. (**b**) Overview of cells in different budding states. (**c**) A cell during cytokinesis, which produced five buds at this pole in previous cytokineses (note the five ring-like bud scars on the surface, indicated by arrows). (**d**) A cell with the typical lemon-shape and bud scars (indicated by arrows) at both poles.

The *Hanseniaspora uvarum* strain used in this work, further designated as HHO1, was previously obtained by selecting an isolate from the German stock collection, DSM 2768, for single-celled growth [31]. The sequence of its diploid genome has largely been determined [31]. A comparison of growth rates in rich medium showed that HHO1 generally grows faster than *Saccharomyces cerevisiae* at temperatures between 15–30 °C (µ = 0.25–0.53 h^−1^ for HHO1, as compared to µ = 0.15–0.45 h^−1^ for *S. cerevisiae* strain HD56-5A), but is more sensitive to warmer conditions, with very poor growth at 37 °C (µ = 0.07 h^−1^ as opposed to µ = 0.35 h^−1^ for HD56-5A), and loss of viability at 45 °C [38]. Regarding their morphology, cells are considerably smaller than those of the baker’s yeast and display the typical, lemon-like shape (Figure 1a; hence the former designation as *Kloeckera apiculata*; [24]). The bipolar budding pattern with consecutive rings of bud scars at both cell poles is more readily observed in scanning electron micrographs (Figure 1b–d).

### 2.2. Cloning and Characterization of Chromosomal Replication Origins

**Figure 2:**
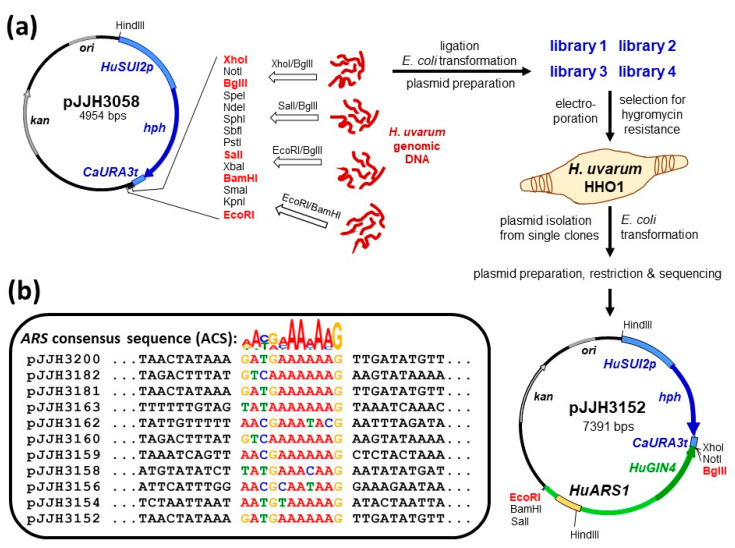
Isolation and characterization of autonomously replicating sequences (*ARS*) from *H. uvarum*. (**a**) Strategy for cloning of *HuARS* elements. Genomic DNA of strain HHO1 was digested with the restriction enzymes indicated and cloned into the respective sites of pJJH3058. The genomic libraries obtained after transformation and amplification in *E. coli* were introduced into HHO1 and plasmids from colonies growing on selective medium with hygromycin B were again isolated and characterized by restriction mapping and sequencing. Protein-coding genes on plasmids are indicated by arrows, regulatory regions, and replication origins by bars. (**b**) Putative ACS-motif of the isolated *ARS*-sequences. The sequences of the inserts of potential *ARS*-carrying plasmids were first searched for *ARS*-sequences using the “ori finder 3” online-tool [39]. Because the identified sequences strongly varied in length (between 70 and 725 bps), the MEME suite 5.5.0 [40] was used to identify a common ACS-motif that is present in all sequences. Note that the *ARS* sequences of the cloning vectors pJJH3181 and pJJH3200, further designated as *HuARS1*, were derived as shorter versions from the original isolate pJJH3152, that of pJJH3182 (*HuARS2*) originated from pJJH3160.

Genetic manipulations of yeasts depend on a method to efficiently introduce homologous or heterologous genes. This is greatly facilitated by the availability of selectable, self-replicating episomal plasmids. To obtain such vectors for propagation in *H. uvarum*, the hygromycin resistance cassette from pJB2 [37] was first cloned into pUK15JJH as an XhoI/HindIII fragment. The resulting plasmid, pJJH3058, was employed to construct genomic libraries for the isolation of replication origins. For this purpose, it was digested in four independent restrictions with XhoI/BglII, SalI/BglII, EcoRI/BglII, and EcoRI/BamHI (Figure 2a).

Genomic DNA obtained from the *H. uvarum* strain HHO1 was digested with the same combination of restriction enzymes, ligated with the respective vector fragments, and transformed into *E. coli*, selecting for kanamycin resistance. Approximately 5000 independent clones were obtained for each library, as judged from colony counts of appropriate dilutions on plates. Cells were washed from plates with the undiluted transformants with 2 mL of LB-kanamycin, each, collected in sterile tubes and used to inoculate fresh overnight cultures in selective medium. From those, plasmid DNA was prepared and introduced into strain HHO1 by electroporation (Figure 2a). Selection on rich medium with hygromycin yielded almost confluent growth on plates obtained from all four libraries, i.e., more than 1000 colonies, each. Three single colonies were picked from the borders of each plate and plasmids were isolated from the twelve overnight cultures grown in rich medium supplemented with 300 mg/L hygromycin B. Clones were readily obtained from all preparations after transformation of *E. coli* and selection for kanamycin resistance and plasmids from single colonies were isolated and subjected to restriction analysis and sequencing. Three clones yielded the same plasmid, designated as pJJH3152, which carried the *GIN4* homologue of *S. cerevisiae* and approximately 1.7 kb of its 5′-flanking sequence (Figure 2a). This and pJJH3160, one of the remaining plasmids carrying *HuSTB5* with its 5′-flanking sequence, were chosen for re-introduction into *H. uvarum* HHO1, to confirm high transformation frequencies on hygromycin medium. Insertions from pJJH3152, pJJH3160 and six further isolates could be assigned to different contigs of the original genomic sequence [31]. We concluded that these plasmids carried autonomously replicating sequences (*ARS*), i.e., chromosomal replication origins, and applied the “ori finder 3” program [39], followed by an analysis through “MEME suite” [40] to determine their location in the inserted *H. uvarum* DNA. As shown in Figure 2b, a consensus sequence could be defined, which consists of 11 bp. To confirm the functionality of the postulated *HuARS* sequences, fragments of 367 bp and 414 bp were obtained by PCR from pJJH3152 and pJJH3160, respectively, and inserted as XhoI/BglII fragments into the original cloning vector pJJH3058. Both resulting plasmids, pJJH3181 (*HuARS1*) and pJJH3182 (*HuARS2*), autonomously replicated in *H. uvarum*, as judged by their high transformation frequencies on hygromycin plates (see Section 2.4).

### 2.3. Construction of Recyclable Deletion Cassettes and Cloning Vectors

As our strain of *H. uvarum* is diploid and two alleles of a given gene would thus generally need to be deleted to obtain a selectable phenotype, it was most unfortunate that only the hygromycin resistance cassette was available as a dominant selection marker [37]. Once used, the resultant strain would be resistant to the antibiotic without a possibility to remove the marker for deletion of the second allele. Moreover, an increase in the hygromycin B concentration to select for deletion of the second allele, as reported for the homozygous knockout of the *HuATF1* gene [37], did not work in our hands with the deletion of genes for auxotrophic markers. To test its suitability, we therefore inserted the G418 resistance cassette from pUG6 [41], which carries the *kanMX* gene flanked by the promoter and terminator of *TEF2* from *Ashbya gossypii*, into pJJH3181 to yield pJJH3197. Transformants first selected on hygromycin plates were also able to grow on plates with G418, confirming that this marker cassette, which is commonly used for deletion of genes in other yeasts, also works for *H. uvarum*. Importantly, it is flanked by *loxP* sites, thus enabling marker recycling upon expression of the gene encoding the bacteriophage P1 Cre-recombinase [42].

**Figure 3:**
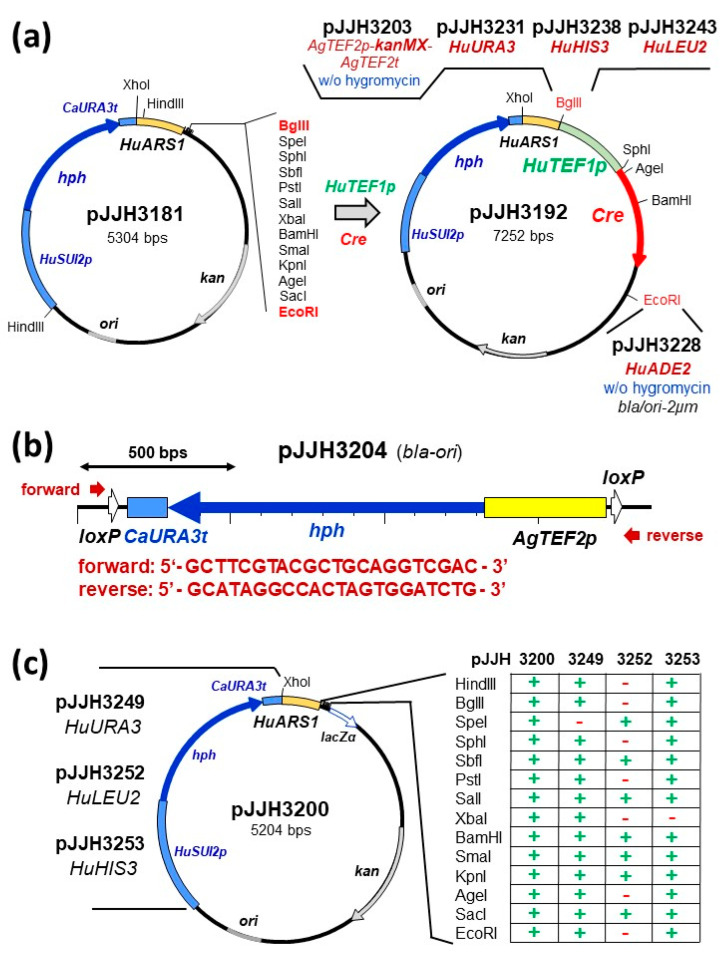
Summary of DNA tools constructed for the genetic manipulation of *H. uvarum*. (**a**) Plasmids for expression of the gene encoding the bacteriophage P1 Cre recombinase. The cloning vector pJJH3181 was derived from the original screen for *HuARS* elements and first used to assemble the strong and constitutive *HuTEF1* promoter with the Cre coding sequence. The resulting plasmid pJJH3192 was then used to introduce the other selectable markers as indicated. While constructs with the auxotrophic markers *HuURA3*, *HuHIS3*, and *HuLEU2* also retained the hygromycin resistance cassette, it was removed from the vector conferring G418 resistance (pJJH3203). Note that the *HuADE2* marker was introduced in a different backbone of the *S. cerevisiae* vector YEp352 [43] and can be selected for ampicillin instead of kanamycin resistance in *E. coli*. (**b**) A hygromycin resistance cassette comprised of sequences entirely heterologous to the *H. uvarum* genome. The cassette was assembled from PCR-generated DNA fragments between the *loxP* sites of an *E. coli* cloning vector derived from pUG6 [41]. The forward and reverse sequences can be added to the 3′-end of longer oligonucleotides providing homologies to the target gene of interest. They are universally applicable to all templates of the pUG-series [41]. *bla-ori* indicates the ß-lactamase gene conferring resistance to ampicillin and the ability to replicate in *E. coli*. (**c**) A set of *H. uvarum/E. coli* shuttle cloning vectors. pJJH3200 was derived from pJJH3181 by restoring the unique HindIII recognition sequence to the multiple cloning site. The hygromycin resistance cassette was then substituted by the different auxotrophic marker genes, as indicated at the left. The table at the right indicates which restriction sites remain unique, i.e., available for cloning, in each vector (+), or those where a second site has been introduced by the marker gene (-).

Consequently, we proceeded by constructing plasmids with the *HuARS1* sequence and different selection markers for production of the Cre-recombinase in *H. uvarum* (Figure 3a). First, pJJH3192 was obtained by simultaneous cloning of two PCR fragments carrying the *HuTEF1* promoter and the coding sequence for the Cre-recombinase into pJJH3181, which can be selected for resistance to hygromycin and constitutively expresses the recombinase gene. Next, pJJH3203 was obtained with the same constitutive expression construct, but the *kanMX* cassette for selection on G418 instead of the hygromycin resistance. Finally, a set of Cre expression plasmids with auxotrophic markers were obtained, comprising pJJH3228 (*HuADE2*), pJJH3231 (*HuURA3*), pJJH3238 (*HuHIS3*), and pJJH3243 (*HuLEU2*). To employ these constructs with a recyclable deletion cassette for the hygromycin resistance marker, we amplified the *TEF2* promoter from *A. gossypii* from pUG6 and the coding sequence of the hygromycin resistance gene from pJB2 for insertion between two *loxP* sites to yield pJJH3204 (Figure 3b). This vector carries the same sequences flanking the deletion cassette as pUG6 and the other plasmids of this series [41], so that the same oligonucleotides can be employed for PCR-based one-step gene replacements with different selection markers.

Antibiotics like G418 and hygromycin are a fairly expensive way of selection in yeast. Based on the autonomously replicating plasmid pJJH3200, we therefore substituted the sequences conferring hygromycin resistance by different auxotrophic markers for *H. uvarum* to generate a set of cloning vectors. Hence, pJJH3249 (*HuURA3*), pJJH3252 (*HuLEU2*), and pJJH3253 (*HuHIS3*) were constructed, with several unique restriction sites, as depicted in Figure 3c. Their applicability is demonstrated in the following Section 2.4.

### 2.4. Construction of Strains with Auxotrophic Selection Markers and Determination of Transformation Efficiencies

**Figure 4:**
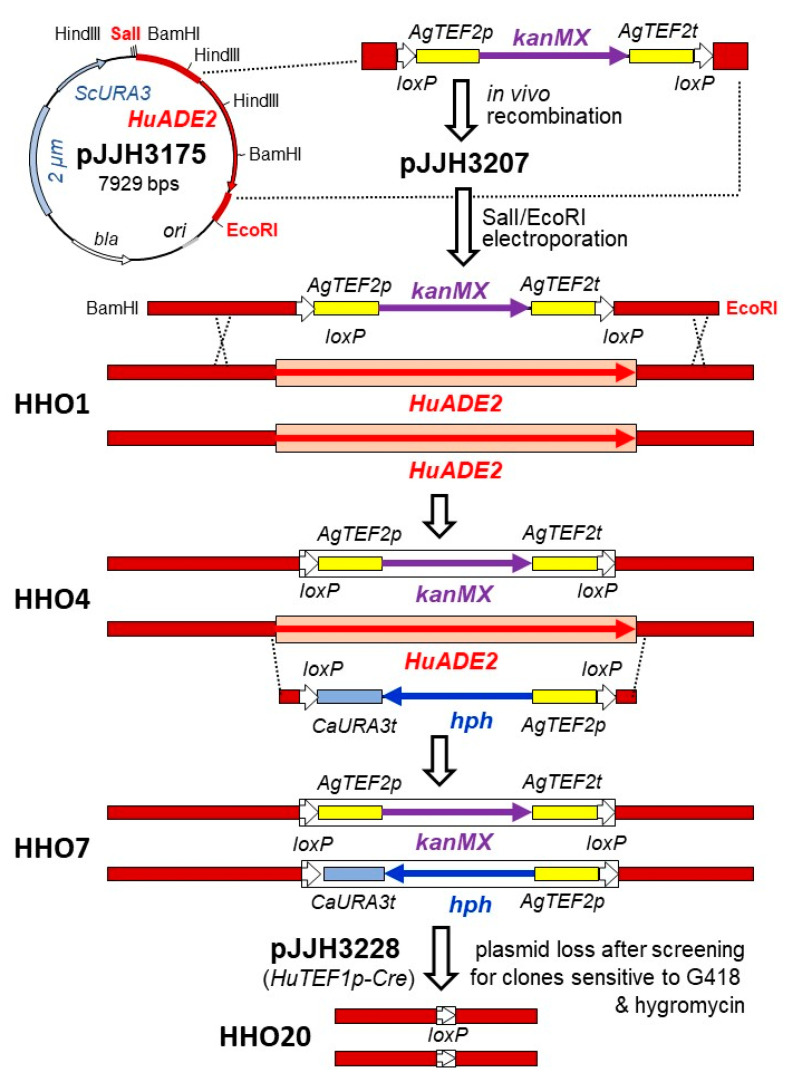
Exemplary strategy for construction of homozygous deletions in *H. uvarum*. The wild-type *HuADE2* gene cloned into the vector YEp352 [43] was introduced for in vivo recombination into *S. cerevisiae* DHD5 together with a PCR-generated deletion cassette, to yield pJJH3207. The latter carries long flanking sequences of the *HuADE2* locus (approximately 800 bp and 150 bp 5′ and 3′ to the coding sequence, respectively). The deletion fragment excised as an EcoRI/BamHI fragment was introduced into *H. uvarum* strain HHO1 by electroporation and colonies grown on selective G418 medium were picked onto a master plate. A clone carrying the respective deletion identified by PCR with flanking primers and confirmed by two further PCRs generating upstream and downstream fragments with primers specific for the deletion cassette, was designated as HHO4 and chosen for the next step. A PCR-generated hygromycin resistance cassette was then used directly to delete the second *HuADE2* allele by homologous recombination, to generate strain HHO7. Introduction of the Cre-recombinase plasmid pJJH3228 selecting for complementation of the adenine auxotrophy finally yielded HHO20, with both alleles retaining a single *loxP* site, each.

In order to show that these constructs are functional in *H. uvarum*, and to obtain a set of recipient strains with auxotrophic selection markers, we proceeded by first deleting the *HuADE2* gene. The coding sequence was substituted for the *kanMX* cassette by in vivo recombination with an episomal plasmid in *S. cerevisiae*, as described in Materials and Methods. The resulting vector, pJJH3207, was digested with EcoRI/BamHI to generate the deletion cassette with large flanking sequences and introduced by electroporation into strain HHO1, selecting for G418 resistance (Figure 4).

Two clones were confirmed by PCR to carry the heterozygous deletion and HHO4 (*Huade2::kanMX/HuADE2*) was used as a recipient for a PCR-generated deletion cassette with the hygromycin resistance marker from pJJH3204. Less than 1% of the colonies obtained displayed poor growth and turned red after one week of incubation. Four of these clones were checked by PCR and shown to contain a homozygous deletion of the two *HuADE2* alleles, one substituted by the *kanMX* and the other by the *hph* genes (Figure 4). As both dominant markers were flanked by *loxP* sites, pJJH3228 (*HuADE2-HuTEF1p-Cre*) was then introduced into strain HHO7 (*Huade2::kanMX/Huade2::hph*) and selected for growth on medium lacking adenine. Fifty colonies were picked onto a master plate on the same medium, incubated over night at 30 °C and replica-plated onto rich medium with either G418 or hygromycin B. All were sensitive to both antibiotics, showing that the Cre recombinase was expressed and efficiently removed the marker cassettes. Subsequent PCR and sequencing confirmed that both *HuADE2* loci carried a single *loxP* site instead of the open reading frame in strain HHO20 (*Huade2::loxP/Huade2::loxP*; Figure 4). By similar approaches, heterozygous and homozygous deletions were obtained for *HuHIS3*, *HuURA3*, and *HuLEU2* (Table 1).

**Figure 5:**
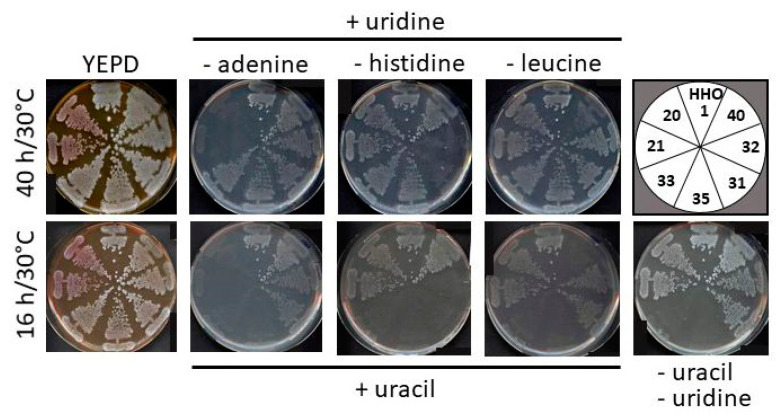
Growth of different auxotrophic mutants on synthetic media. Cells were streaked out for single colonies on standard rich medium (YEPD) and allowed to grow over night at 30 °C. They were then replica-plated onto different drop-out media and incubated for the times indicated. Strain designations in the upper right corner correspond to those listed in Table 1. Uridine and uracil were added as indicated at final concentrations of 1.7 mM and 0.2 mM, respectively.

For all further deletions, except *Huhis3::kanMX*, the PCR generated marker cassettes were directly employed for transformation of *H. uvarum*, without prior addition of longer flanking homologies by in vivo recombination in *S. cerevisiae*. Further, in the case of *HuURA3* and *HuLEU2* the hygromycin resistance cassette generated from pJJH3204 was exclusively employed, with removal from the heterozygous deletions prior to deletion of the second allele with the same PCR product. It should also be noted that the efficiency of the desired homologous recombination was low, generally yielding correct replacements in less than 3% of all clones examined. In contrast, once substituted by the selection markers, homozygous deletions leaving only *loxP* sites in both alleles of the gene in question were readily obtained by introducing one of the plasmids for Cre recombinase constructed in Section 2.3. More than 95% of clones grown on a master plate selecting for maintenance of the Cre plasmid had lost the deletion markers upon replica-plating onto the respective antibiotics (G418 and/or hygromycin). Subsequently, non-selective growth in liquid rich medium overnight and streaking out for single colonies on plates yielded more than 80% of clones that also had lost the plasmid. Figure 5 shows the growth of the different homozygous deletion mutants on drop-out media, confirming the auxotrophic requirements. It should be noted that *Huura3::loxP/Huura3::loxP* mutants failed to grow on all synthetic media, even if supplemented with uracil, but did grow when uridine was added, instead. The auxotrophic mutants may thus serve as recipients for further manipulations, i.e., for introduction of heterologous genes on the vectors carrying the respective wild-type genes from *H. uvarum* (Figure 3c).

### 2.5. Establishment of a Chemical Transformation Procedure

During these studies, electroporation proved to be very efficient and reliable to introduce deletion cassettes but is fairly laborious and expensive for the introduction of episomal plasmids. We therefore investigated whether (i) competent cells can be stored for electroporation, and (ii) if they can be employed for an alternative transformation protocol. As shown in Table 2, electroporation of fresh competent cells with episomal plasmids yielded transformation frequencies in the order of 10^5^ transformants per µg of DNA. This frequency was only reduced by half after storage of the electrocompetent cells for up to 17 days at −80 °C, which is more than sufficient for the introduction of episomal plasmids.

Employing these stored electrocompetent cells in a variation of the freeze-transformation method by [44] resulted in similar transformation frequencies in the range of 10^5^ transformants per µg of plasmid DNA (Table 2), which were also observed for episomal plasmids in the respective auxotrophic recipient strains (Table 2), permitting long-term storage and fast transformation, if the same recipient strain is to be used for various manipulations.

## 3. Discussion

This work was initiated with the aim of providing a useful set of strains, plasmids, and deletion cassettes for the genetic manipulation of *Hanseniaspora uvarum*. In this context, one of the key achievements was the isolation and tailoring of autonomously replicating sequences (*ARS*), i.e., chromosomal replication origins. These were readily obtained from genomic libraries by their ability to confer relatively high transformation frequencies, with approximately 10^5^ transformants per µg of plasmid DNA. While that is still at least two orders of magnitude lower than those routinely obtained with similar plasmids in *Saccharomyces cerevisiae* [45], it clearly allows the fast and easy introduction of either homologous or heterologous genes for expression in *H. uvarum*. The *ARS* consensus sequence (ACS) defined herein (Figure 2b) differs considerably from that of *S. cerevisiae* [46], explaining why previous attempts to introduce episomal vectors from this yeast failed (this laboratory, unpublished results). Besides an ACS, functional *ARS* elements in *S. cerevisiae* also comprise at least a B1 and a B2 region with even less sequence conservation, together spanning approximately 100 base pairs [46]. As we located a functional *HuARS1* sequence on a 367 bp fragment, we decided that this suffices for all practical purposes and did not do further molecular analyses.

Instead, as a proof-of-principle and to facilitate the multiple use of deletion markers, we cloned the bacteriophage P1 Cre-recombinase coding sequence on such an episomal plasmid, expressed from the *HuTEF1* promoter. The Cre-recombinase has been widely employed in the construction of deletion mutants in the model yeasts *S. cerevisiae* and *Kluyveromyces lactis* to eliminate target genes flanked by direct repeats of short *loxP* sequences [42,47]. It also worked very efficiently in *H. uvarum*, as demonstrated by the fact that almost all clones growing on selective medium for the maintenance of the *Cre* expression plasmid had lost the respective deletion marker cassettes by the time they formed colonies and were transferred to a master plate. We attribute this behaviour to the fact that the *HuTEF1* promoter allows for high constitutive expression of the recombinase gene, as it drives the expression of the gene encoding a translational elongation factor required in high quantities at its native locus. Moreover, plasmids carrying *ARS* sequences are known to reside in high copy numbers and are preferentially retained in the mother cells in *S. cerevisiae*, in fact constituting a cause of aging when carrying ribosomal DNA genes [48,49]. The mitotic instability of such *ARS* plasmids is presumably conserved in *H. uvarum*, as the Cre-recombinase constructs were readily lost within one overnight growth in non-selective medium, further facilitating the use of this system.

We here demonstrated that gene deletions can be obtained by introducing PCR-generated, *loxP*-flanked dominant selection markers. Two features were improved on the previously reported use of hygromycin B resistance [37]: (i) Besides the addition of flanking *loxP* sites, the *hph* gene encoding the hygromycin phosphatase was placed under the control of the heterologous *AgTEF2* promoter from *Ashbya gossypii*, instead of the *HuSUI2* promoter, to minimize the unwanted integration of circularized PCR products at the native locus. (ii) The commonly used *kanMX* cassette residing between the *AgTEF2* promoter and terminator sequences, and flanked by *loxP* sites [50], was shown to work as a second dominant selection marker in *H. uvarum*. As the pUG6 vector used as a template to generate this cassette is part of a series also carrying heterologous marker genes for complementation of *his3*, *leu2*, and *ura3* mutants in *S. cerevisiae* [41], we expect that these can be exploited in future studies for gene deletions using the auxotrophic mutants generated here, with the advantage that all can be amplified by PCR with one pair of oligonucleotide primers.

As expected for the auxotrophic markers, the deletion of one allele from the diploid genome did not result in a notable phenotype, but homozygous deletions failed to grow on the respective drop-out media. The *Huade2* mutants are especially of interest in this context, as they accumulate a red pigment, so that genetic screens for colony colour and sectoring can be used to assess the stability of plasmids and chromosomes equipped with the wild-type *HuADE2* gene, similar to *S. cerevisiae* [51]. For instance, this could be employed to isolate centromeric sequences from the *H. uvarum* genome, similar to the isolation of *HuARS* sequences from libraries as described herein.

The homozygous *Huura3* deletions also offer a range of future applications, for example, counter-selection of plasmids carrying the *HuURA3* gene will be possible on medium with 5-fluoro-acetate (5-FOA), allowing for plasmid-shuffling [52]. In fact, we previously isolated *Huura3* mutants from an aneuploid strain induced by nocodazole, based on its resistance to 5-FOA [31]. A puzzling result then was that it not only failed to grow on medium lacking uracil, but generally on synthetic medium. This feature is shared by the homozygous deletion obtained here and could be explained by a similar metabolism to *A. gossypii*. There, *Agura3* mutants were also found to be sensitive to the presence of uracil in synthetic media, leading to inhibition of the uracil phosphoribosyltransferase reaction in the pyrimidine salvage pathway [53]. As observed in that work, growth could be restored in *Huura3* mutants by substituting uracil for uridine.

The heterozygous deletion mutants listed in Table 1 may also be of value in future studies. One may want to stably integrate heterologous genes into the *H. uvarum* genome, e.g., to increase the sulphite resistance for improved use in must fermentations. Using an internal fragment of the coding sequence, e.g., of the *HuURA3* gene, on a non-replicating plasmid, an integration into the remaining allele in the heterozygous mutant (*Huura3::loxP/HuURA3*) would result in an auxotrophy for uracil selectable on 5-FOA. In addition, the low efficiency of homologous recombination could be improved by diminishing the activity of the non-homologous end joining repair, e.g., deleting the two copies of the *HuKU80* gene, as proven valuable in *K. lactis* [54]. Alternatively, taking advantage of both the high expression levels and the mitotic instability of the *HuARS* vectors described here, *ScRAD51*, a component of the homologous repair system of *S. cerevisiae*, could be transiently over-expressed from the *HuTEF1* promoter in co-transformations with deletion cassettes, as exercised in lager yeast [55].

As examplified by these few remarks, the toolset provided in this work allows a wealth of future applications for *H. uvarum*, which can be employed not only to improve its performance in the production of alcoholic beverages like wine and cider, but also in its use as a biocontrol agent in fighting fungal infections, insect pests, or enhance the growth of agricultural plants. Clearly, a deeper understanding of its basic metabolic capacities lies at the heart of such applications, which may also be broadened with the molecular genetic techniques now at hand. Finally, given the close phylogenetic relationship with other *Hanseniaspora/Kloeckera* varieties [36], the tools developed herein may probably also be of use for the genetic manipulation of other members of the genera.

## 4. Materials and Methods

### 4.1. Strains, Media, and Growth Conditions

All strains of *Hanseniaspora uvarum* applied and constructed in this work are based on HHO1, which is derived from strain DSM 2768, also designated as CBS 2584 or NRRL 115. It was obtained from the German stock culture collection and selected for single-celled growth, as described previously [31]. *Saccharomyces cerevisiae* strains HD56-5A and its diploid derivative DHD5 have been described [56,57]. Yeast cell culture and genetic techniques followed standard procedures devised for *S. cerevisiae* [58]. Rich medium (YEPD) contained 1% yeast extract, 2% Bacto peptone (Difco Laboratories Inc., Detroit, MI, USA), and 2% glucose. Synthetic media were prepared as described in [58], with the omission of amino acids or bases as required for selection of plasmids or deletion markers and 2% glucose (SCD). If required, uracil was substituted for 1.7 mM uridine. For selection of the *kanMX* marker, 50 mg/L of G418 were added to the rich medium after sterilization, for selection of hygromycin resistance, 300 mg/L of hygromycin B were employed (both antibiotics were purchased from Carl Roth GmbH, Karlsruhe, Germany).

For manipulations in *E. coli*, strain DH5α was employed with standard media as described in [59].

### 4.2. Transformation Procedures

For electroporation, the method described by [37] was adopted and modified as follows: To prepare five aliquots of competent cells, a pre-culture of *H. uvarum* was inoculated in 3 mL of YEPD and incubated with shaking over night at 30 °C. The culture was diluted 1:30 in fresh YEPD and 5 µL were used to inoculate 50 mL of YEPD in a 300 mL Erlenmeyer flask. After shaking for 16 h at 180 rpm at 30 °C the culture reached an OD_600_ of 3.5–4.0, determined with a Beckman spectrophotometer DU800 (Beckman Coulter, Krefeld, Germany). If higher densities were observed, cells were diluted again to an OD_600_ of 1.0 and incubated for approximately 2 h until they reached the required final OD_600_. Cells were collected in a sterile Falcon tube by centrifugation for 5 min at 1700× *g* at room temperature and resuspended in 25 mL of sterile water. After another centrifugation step (5 min/1700× *g*) the pellet was resuspended in 25 mL of lithium acetate buffer (10 mM Tris, pH 8.0, 1 mM EDTA, 100 mM lithium acetate, 10 mM dithiothreitol), with the components freshly mixed from 10-fold concentrated stock solutions. After incubation for 1 h at room temperature, cells were collected by centrifugation, washed with 25 mL of ice-cold sterile water, and resuspended in 20 mL of cold sterile 1 M sorbitol. Cells were again pelleted, resuspended in 500 µL of cold sterile 1 M sorbitol and aliquoted in 100 µL samples into sterile electroporation cuvettes (Gene Pulser Cuvette 0.2 cm electrode gap from BioRad/Feldkirchen/Germany). Alternatively, 100 µL aliquots of the sorbitol suspension were stored in sterile Eppendorf tubes at −80 °C and directly used for electroporation after thawing on ice. For electroporation, 10 µL of plasmid DNA, or 20 µL of PCR product or digested DNA purified with Roche High-Pure PCR purification kit (Roche/Mannheim/Germany), were added to the cuvettes at room temperature and mixed well (note that products from restriction digests or PCR reactions cannot be applied directly for electroporation without damaging the cuvettes). After an incubation of 5–10 min at room temperature, electroporation was performed with an Electro Cell Porator 600 (BTX Electroporation Systems, Fisher Scientific GmbH, Schwerte, Germany). A program designed for electroporation of *E. coli* cells was applied, with 1.55 kV for 5.18 msec at a resistance of 2.5 kV. Cuvettes were then kept on ice and 500 µL of ice-cold sterile 1M sorbitol were added. The cell suspension was transferred into 1.5 mL of fresh YEPD medium and either incubated with shaking at 30 °C for 3–4 h for regeneration when dominant markers (G418 or hygromycin B) were employed, or 100 µL were directly plated onto selective media in the case of auxotrophic markers (*HuADE2, HuURA3, HuHIS3, HuLEU2*), from appropriate dilutions, if required.

For introduction of episomal plasmids, a chemical transformation procedure was developed. It followed the preparation of competent cells described above for electroporation, except that 200 µL aliquots of the sorbitol suspension prior to electroporation were stored at −80 °C until use. For DNA addition and further treatment, the freeze-transformation method [44] was modified. Thus, 5 µL of herring-sperm DNA (10 µg/µL from Roche, Mannheim, Germany) were added as carrier DNA to the frozen cells, together with 10–20 µL of plasmid solution, corresponding to 0.1–0.5 µg of DNA. Cells were thawed and heat-shocked with occasional shaking at 32 °C, and 500 µL of solution B (40% polyethylene glycol 1000, 100 mM bicine pH 8.35) were added. After an incubation for 1 h at 28 °C, cells were collected by centrifugation (2 min at 13,000× *g*), the supernatant discarded, and the pellet resuspended in 500 µL of YEPD. One hundred microliters from appropriate dilutions (usually 1:50 to 1:200) were then either plated directly onto selective media for auxotrophic markers or regenerated for 4 h with shaking at 30 °C in 2 mL of YEPD in case of selection for antibiotic resistance.

### 4.3. Construction of Plasmids and Deletion Mutants

The basic vectors employed were derived from pUK15JJH, a variant of pUK1921 [60], with an extended multiple cloning site, and pJB2, a plasmid for selection of hygromycin resistant deletions reported by [37], (note that the resistance gene is under the control of the *HuSUI2* promoter instead of the *HuTEF1* promoter, as originally reported). One-step gene replacements [61] were either obtained by transformation of marker cassettes with long flanking sequences homologous to the target loci and excised from plasmids with appropriate restriction enzymes, or directly by the introduction of PCR products with flanking homologies of 50 ± 7 bais pairs. For cloning in *S. cerevisiae*, standard vectors were used [43,62]. The *kanMX* deletion cassette was derived from pUG6 [41], which was also used as a source for the *AgTEF2* promoter. Restriction enzymes were obtained from Thermo Fisher Scientific, Schwerte, Germany, and used according to the manufacturer’s instructions. Plasmids were generated either by standard restriction-ligation reactions from previously cloned DNA or PCR products, or by in vivo recombination in *S. cerevisiae*. Details on the manufacture of specific constructs, maps, and sequences of all plasmids and genomic loci with the deletion constructs are available upon request. Oligonucleotides used as primers for cloning, deletions and PCR verifications can be found in Supplementary Table S1. All PCR-generated fragments further used in cloning and expression studies were verified by Sanger sequencing (Seqlab, Göttingen, Germany).

### 4.4. DNA Preparations and PCR

Genomic DNA from *H. uvarum* was prepared by a rapid, phenol-free extraction procedure as follows: 500 µL of a late-logarithmic overnight culture in YEPD were collected by centrifugation in a 1.5 mL Eppendorf tube for 1 min at 10,000× *g* and resuspended in 500 µL of spheroblast buffer (0.9 M sorbitol, 0.1 M EDTA, pH 8.0). Next, 2 µL of a Zymolyase stock solution (10 mg/mL of Zymolyase 20T stored at −20 °C) and 10 µL of a 100 mM stock solution of DTT were added and the cells were incubated for 15 min at 37 °C. Spheroblasts were collected by centrifugation for 1 min at 10,000× *g* and the pellet was suspended in 300 µL TE/SDS (12.5 mM Tris pH 7.5, 5 mM EDTA, 0.25% SDS). The suspension was incubated for 10 min at 65 °C to inactivate DNases, transferred to ice and 80 µL of a 5 M potassium acetate solution were added. After incubation for 5 min, the tubes were centrifuged for 10 min at 10,000× *g* and the 350 µL of the supernatant was removed into fresh Eppendorf tubes. Then, 800 µL of pure ethanol were added for precipitation of the genomic DNA, mixed well, and subjected to centrifugation (10 min at 10,000× *g*). The pellet was dried and dissolved in 100 µL of an RNase solution (solution 1 from the High Pure Plasmid Purification kit from Roche, Mannheim, Germany, supplemented with 150 mM sodium acetate, pH 7.0, to avoid DNA nicking activity). Tubes were incubated for 10 min at 37 °C with occasional vigorous shaking. The DNA was again precipitated by addition of 5 µL from a 3 M stock solution of sodium acetate and 250 µL of pure ethanol, followed by 5 min of centrifugation at 10,000× *g*. The pellet was again dried and dissolved in 100 µL of the column elution buffer from the plasmid preparation kit. Finally, 1 µL was then applied to PCR reactions or 10 µL for digestions with restriction enzymes.

Plasmids were isolated from *H. uvarum* by disruption with glass beads, purification through the Roche High-Pure PCR purification kit, followed by transformation into *E. coli* DH5α for amplification, as described for *S. cerevisiae* in a previous work [63].

The PCR reactions were routinely performed in a volume of 50 µL in 0.2 µL Eppendorf tubes in a thermocycler (Biometra GmbH, Göttingen, Germany). For analytical purposes, i.e., confirmation of deletion constructs, the Dream Taq preparation (Thermo Fisher Scientific, Schwerte, Germany) was used according to the instructions of the manufacturer. For cloning purposes, the High Fidelity Taq Polymerase from Roche (Mannheim, Germany) was employed. Custom-made oligonucleotides were used as primers for sequencing and the PCR were purchased from Biolegio BV, Nijmegen, The Netherlands. For sequences of *H. uvarum*, the annotated genome sequence was consulted ([31]; deposited in GenBank at https://www.ncbi.nlm.nih.gov/GenBank/under accession number APLS01000000; accessed on 30 December 2022).

### 4.5. Imaging

Brightfield micrographs were obtained from cultures growing logarithmically in synthetic complete medium with a Zeiss Axioplan 2 microscope (Carl Zeiss, Jena, Germany) with differential interference contrast (DIC) as detailed in [64].

For scanning electron microscopy, the *H. uvarum* strain HHO1 was grown in 3 mL liquid rich medium (YEPD) overnight at 30 °C with shaking, and the late logarithmic culture was used to inoculate 3 mL of synthetic complete medium at an OD_600_ of approximately 0.3 and grown for another four hours into the logarithmic phase. Cells were harvested and treated for imaging exactly as described for *Kluyveromyces lactis* in [65].

## Figures and Tables

**Table 1:** Heterozygous and homozygous deletions constructed in *H. uvarum* HHO1.

Strain	Relevant Genotype	Requirements
HHO4	*Huade2::kanMX/HuADE2*	none
HHO12	*Huade2::loxP/HuADE2*	none
HHO7	*Huade2::kanMX/Huade2::hph*	adenine
HHO20/HHO21	*Huade2::loxP/Huade2::loxP*	adenine
HHO2	*Huhis3::kanMX/HuHIS3*	none
HHO17	*Huhis3::loxP/HuHIS3*	none
HHO14	*Huhis3::kanMX/Huhis3::hph*	histidine
HHO31/HHO32	*Huhis3::loxP/Huhis3::loxP*	histidine
HHO25	*Huleu2::hph/HuLEU2*	none
HHO37	*Huleu2::loxP/HuLEU2*	none
HHO40	*Huleu2::loxP/Huleu2::hph*	leucine
HHO44	*Huleu2::loxP/Huleu2::loxP*	leucine
HHO6	*Huura3::hph/HuURA3*	none
HHO19	*Huura3::loxP/HuURA3*	none
HHO29	*Huura3::loxP/Huura3::hph*	uridine ^1^
HHO33/HHO35	*Huura3::loxP/Huura3::loxP*	uridine ^1^

^1^ note that these strains do not grow on synthetic media supplemented with uracil, but require uridine, instead (also see Figure 5); growth on rich medium (YEPD) is not impaired.

**Table 2:** Transformation frequencies obtained with different plasmids and procedures.

Strain	Procedure ^1^	Plasmid ^2^	Selection	Transformants/µg ^3^
HHO1	EP	pJJH3181	hygromycin	5.0 × 10^5^
HHO1	EP	pJJH3182	hygromycin	2.4 × 10^5^
HHO1	EP	pJJH3200	hygromycin	5.0 × 10^5^
HHO1	FM	pJJH3200	hygromycin	4.0 × 10^5^
HHO21	FM	pJJH3228	SC w/o adenine	1.3 × 10^5^
HHO31	EP	pJJH3253	SC w/o histidine	0.2 × 10^5^
HHO31	FM	pJJH3253	SC w/o histidine	0.5 × 10^5^
HHO40	FM	pJJH3252	SC w/o leucine	1.8 × 10^5^
HHO35	FM	pJJH3259	SC w/o uracil	6.0 × 10^5^

^1^ EP = electroporation; FM = freeze method. ^2^ plasmids all carry an *HuARS* sequence and are described in the text and Figure 3c. ^3^ transformants per µg of plasmid DNA were calculated from colony counts on selective media, after transformation with 0.1–0.5 µg of DNA in a volume of 10–20 µL.

## Data Availability

Not applicable.

## Supplementary Materials

The supporting information can be downloaded at: https://www.mdpi.com/article/10.3390/ijms24031859/s1.

## Abbreviations

ARS, Autonomously Replicating Sequence; bp, base pairs; DIC, Differential Interference Phase Contrast; EDTA, Ethylene Diamine Tetra Acetic acid; EP, electroporation; FM, Freeze Method; LB, Luria Broth; OD_600_, Optical Density at 600 nm; PCR, Polymerase Chain Reaction; PEG, Polyethylenglycol; SCD, Synthetic Complete Dextrose medium; SDS, sodium dodecyl sulfate; SEM, Scanning Electron Microscopy; YEPD, Yeast Extract Peptone Dextrose medium.

## References

[1] Chambers P.J., Pretorius I.S. (2010). Fermenting knowledge: The history of winemaking, science and yeast research. EMBO Rep..

[2] Drumonde-Neves J., Franco-Duarte R., Lima T., Schuller D., Pais C. (2016). Yeast biodiversity in vineyard environments is increased by human intervention. PLoS ONE.

[3] Fleet G.H. (2003). Yeast interactions and wine flavour. Int. J. Food Microbiol..

[4] Tufariello M., Fragasso M., Pico J., Panighel A., Castellarin S.D., Flamini R., Grieco F. (2021). Influence of non-*Saccharomyces* on wine chemistry: A focus on aroma-related compounds. Molecules.

[5] Kachalkin A., Glushakova A., Streletskii R. (2022). Diversity of endophytic yeasts from agricultural fruits positive for phytohormone IAA production. BioTech.

[6] Liu Z., Tian J., Yan H., Li D., Wang X., Liang W., Wang G. (2022). Ethyl acetate produced by *Hanseniaspora uvarum* is a potential biocontrol agent against tomato fruit rot caused by *Phytophthora nicotianae*. Front. Microbiol..

[7] Skotniczny M., Satora P., Panczyszyn K., Cioch-Skoneczny M. (2020). Growth dynamics and diversity of yeasts during spontaneous plum mash fermentation of different varieties. Foods.

[8] Wang L., Dou G., Guo H., Zhang Q., Qin X., Yu W., Jiang C., Xiao H. (2019). Volatile organic compounds of *Hanseniaspora uvarum* increase strawberry fruit flavor and defense during cold storage. Food Sci. Nutr..

[9] Batista N.N., Ramos C.L., Dias D.R., Pinheiro A.C., Schwan R.F. (2016). The impact of yeast starter cultures on the microbial communities and volatile compounds in cocoa fermentation and the resulting sensory attributes of chocolate. J. Food Sci. Technol..

[10] Masoud W., Cesar L.B., Jespersen L., Jakobsen M. (2004). Yeast involved in fermentation of *Coffea arabica* in East Africa determined by genotyping and by direct denaturating gradient gel electrophoresis. Yeast.

[11] Gomez-Albarran C., Melguizo C., Patino B., Vazquez C., Gil-Serna J. (2021). Diversity of mycobiota in Spanish grape berries and selection of *Hanseniaspora uvarum* U1 to prevent mycotoxin contamination. Toxins.

[12] Liu H.M., Guo J.H., Cheng Y.J., Liu P., Long C.A., Deng B.X. (2010). Inhibitory activity of tea polyphenol and *Hanseniaspora uvarum* against *Botrytis cinerea* infections. Lett. Appl. Microbiol..

[13] Tejero P., Martin A., Rodriguez A., Galvan A.I., Ruiz-Moyano S., Hernandez A. (2021). In vitro biological control of *Aspergillus flavus* by *Hanseniaspora opuntiae* L479 and *Hanseniaspora uvarum* L793, producers of antifungal volatile organic compounds. Toxins.

[14] Zhang Q.Q., Shi J., Shen P.Y., Xi F., Qian C.Y., Zhang G.H., Zhu H.J., Xiao H.M. (2022). Exploring the efficacy of biocontrol microbes against the fungal pathogen *Botryosphaeria dothidea* JNHT01 isolated from fresh walnut fruit. Foods.

[15] Little C.M., Chapman T.W., Hillier N.K. (2020). Plasticity is key to success of *Drosophila suzukii* (Diptera: Drosophilidae) invasion. J. Insect Sci..

[16] Jones R., Fountain M.T., Andreani N.A., Gunther C.S., Goddard M.R. (2022). The relative abundances of yeasts attractive to *Drosophila suzukii* differ between fruit types and are greatest on raspberries. Sci. Rep..

[17] Kleman I., Rehermann G., Kwadha C.A., Witzgall P., Becher P.G. (2022). *Hanseniaspora uvarum* attracts *Drosophila suzukii* (Diptera: Drosophilidae) with high specificity. J. Econ. Entomol..

[18] Schmitt M.J., Poravou O., Trenz K., Rehfeldt K. (1997). Unique double-stranded RNAs responsible for the anti-*Candida* activity of the yeast *Hanseniaspora uvarum*. J. Virol..

[19] Jolly N.P., Varela C., Pretorius I.S. (2014). Not your ordinary yeast: Non-*Saccharomyces* yeasts in wine production uncovered. FEMS Yeast Res..

[20] Zott K., Miot-Sertier C., Claisse O., Lonvaud-Funel A., Masneuf-Pomarede I. (2008). Dynamics and diversity of non-*Saccharomyces* yeasts during the early stages in winemaking. Int. J. Food Microbiol..

[21] Borren E., Tian B. (2020). The important contribution of non-*Saccharomyces* yeasts to the aroma complexity of wine: A review. Foods.

[22] Ciani M., Beco L., Comitini F. (2006). Fermentation behaviour and metabolic interactions of multistarter wine yeast fermentations. Int. J. Food Microbiol..

[23] Huang M., Liu X., Li X., Sheng X., Li T., Tang W., Yu Z., Wang Y. (2022). Effect of *Hanseniaspora uvarum*-*Saccharomyces cerevisiae* mixed fermentation on aroma characteristics of *Rosa roxburghii* Tratt, blueberry, and plum wines. Molecules.

[24] Martin V., Valera M.J., Medina K., Boido E., Carrau F. (2018). Oenological impact of the *Hanseniaspora/Kloeckera* yeast genus on wines—A review. Fermentation.

[25] Pietrafesa A., Capece A., Pietrafesa R., Bely M., Romano P. (2020). *Saccharomyces cerevisiae* and *Hanseniaspora uvarum* mixed starter cultures: Influence of microbial/physical interactions on wine characteristics. Yeast.

[26] Nisiotou A., Mallouchos A., Tassou C., Banilas G. (2019). Indigenous yeast interactions in dual-starter fermentations may improve the varietal expression of Moschofilero wine. Front. Microbiol..

[27] Canonico L., Comitini F., Oro L., Ciani M. (2016). Sequential fermentation with selected immobilized non-*Saccharomyces* yeast for reduction of ethanol content in wine. Front. Microbiol..

[28] Zhu X., Navarro Y., Mas A., Torija M.J., Beltran G. (2020). A rapid method for selecting non-*Saccharomyces* strains with a low ethanol yield. Microorganisms.

[29] Bezerra-Bussoli C., Baffi M.A., Gomes E., Da-Silva R. (2013). Yeast diversity isolated from grape musts during spontaneous fermentation from a Brazilian winery. Curr. Microbiol..

[30] Venturin C., Boze H., Moulin G., Galzy P. (1995). Glucose metabolism, enzymic analysis and product formation in chemostat culture of Hanseniaspora uvarum. Yeast.

[31] Langenberg A.K., Bink F.J., Wolff L., Walter S., von Wallbrunn C., Grossmann M., Heinisch J.J., Schmitz H.P. (2017). Glycolytic functions are conserved in the genome of the wine yeast *Hanseniaspora uvarum*, and pyruvate kinase limits its capacity for alcoholic fermentation. Appl. Environ. Microbiol..

[32] Mencher A., Morales P., Valero E., Tronchoni J., Patil K.R., Gonzalez R. (2020). Proteomic characterization of extracellular vesicles produced by several wine yeast species. Microb. Biotechnol..

[33] Masneuf-Pomarede I., Bely M., Marullo P., Albertin W. (2015). The genetics of non-conventional wine yeasts: Current knowledge and future challenges. Front. Microbiol..

[34] Guaragnella N., Chiara M., Capece A., Romano P., Pietrafesa R., Siesto G., Manzari C., Pesole G. (2019). Genome sequencing and comparative analysis of three *Hanseniaspora uvarum* indigenous wine strains reveal remarkable biotechnological potential. Front. Microbiol..

[35] Sternes P.R., Lee D., Kutyna D.R., Borneman A.R. (2016). Genome sequences of three species of *Hanseniaspora* isolated from spontaneous wine fermentations. Genome Announc..

[36] Saubin M., Devillers H., Proust L., Brier C., Grondin C., Pradal M., Legras J.L., Neuveglise C. (2019). Investigation of genetic relationships between *Hanseniaspora* species found in grape musts revealed interspecific hybrids with dynamic genome structures. Front. Microbiol..

[37] Badura J., van Wyk N., Brezina S., Pretorius I.S., Rauhut D., Wendland J., von Wallbrunn C. (2021). Development of genetic modification tools for *Hanseniaspora uvarum*. Int. J. Mol. Sci..

[38] Bink F.J. (2010). Molekulargenetische und physiologische Untersuchungen an der Weinhefe *Kloeckera apiculata* (*Hanseniaspora uvarum*). Ph.D. Thesis.

[39] Wang D., Lai F.L., Gao F. (2021). Ori-Finder 3: A web server for genome-wide prediction of replication origins in *Saccharomyces cerevisiae*. Brief Bioinform..

[40] Bailey T.L., Johnson J., Grant C.E., Noble W.S. (2015). The MEME Suite. Nucleic Acids Res..

[41] Gueldener U., Heinisch J., Koehler G.J., Voss D., Hegemann J.H. (2002). A second set of *loxP* marker cassettes for Cre-mediated multiple gene knockouts in budding yeast. Nucleic Acids Res..

[42] Hegemann J.H., Heick S.B. (2011). Delete and repeat: A comprehensive toolkit for sequential gene knockout in the budding yeast *Saccharomyces cerevisiae*. Methods Mol. Biol..

[43] Hill J.E., Myers A.M., Koerner T.J., Tzagoloff A. (1986). Yeast/*E. coli* shuttle vectors with multiple unique restriction sites. Yeast.

[44] Klebe R.J., Harriss J.V., Sharp Z.D., Douglas M.G. (1983). A general method for polyethylene-glycol-induced genetic transformation of bacteria and yeast. Gene.

[45] Gietz R.D., Schiestl R.H., Willems A.R., Woods R.A. (1995). Studies on the transformation of intact yeast cells by the LiAc/SS-DNA/PEG procedure. Yeast.

[46] Liachko I., Youngblood R.A., Keich U., Dunham M.J. (2013). High-resolution mapping, characterization, and optimization of autonomously replicating sequences in yeast. Genome Res..

[47] Rodicio R., Heinisch J.J. (2013). Yeast on the milky way: Genetics, physiology and biotechnology of *Kluyveromyces lactis*. Yeast.

[48] Murray A.W., Szostak J.W. (1983). Pedigree analysis of plasmid segregation in yeast. Cell.

[49] Sinclair D.A., Guarente L. (1997). Extrachromosomal rDNA circles--a cause of aging in yeast. Cell.

[50] Wach A., Brachat A., Pohlmann R., Philippsen P. (1994). New heterologous modules for classical or PCR-based gene disruptions in *Saccharomyces cerevisiae*. Yeast.

[51] Hieter P., Mann C., Snyder M., Davis R.W. (1985). Mitotic stability of yeast chromosomes: A colony color assay that measures nondisjunction and chromosome loss. Cell.

[52] Haase M.A.B., Truong D.M., Boeke J.D. (2019). Superloser: A plasmid shuffling vector for *Saccharomyces cerevisiae* with exceedingly low background. G3.

[53] Silva R., Aguiar T.Q., Oliveira C., Domingues L. (2019). Physiological characterization of a pyrimidine auxotroph exposes link between uracil phosphoribosyltransferase regulation and riboflavin production in *Ashbya gossypii*. Nat. Biotechnol..

[54] Heinisch J.J., Buchwald U., Gottschlich A., Heppeler N., Rodicio R. (2010). A tool kit for molecular genetics of *Kluyveromyces lactis* comprising a congenic strain series and a set of versatile vectors. FEMS Yeast Res..

[55] Bernardi B., Kayacan Y., Akan M., Wendland J. (2019). Overexpression of RAD51 enables PCR-based gene targeting in lager yeast. Microorganisms.

[56] Arvanitidis A., Heinisch J.J. (1994). Studies on the function of yeast phosphofructokinase subunits by in vitro mutagenesis. J. Biol. Chem..

[57] Kirchrath L., Lorberg A., Schmitz H.P., Gengenbacher U., Heinisch J.J. (2000). Comparative genetic and physiological studies of the MAP kinase Mpk1p from *Kluyveromyces lactis* and *Saccharomyces cerevisiae*. J. Mol. Biol..

[58] Rose M.D., Winston F., Hieter P. (1990). Methods in Yeast Genetics—A Laboratory Course Manual.

[59] Straede A., Heinisch J.J. (2007). Functional analyses of the extra- and intracellular domains of the yeast cell wall integrity sensors Mid2 and Wsc. FEBS Lett..

[60] Heinisch J.J. (1993). *PFK2, ISP42, ERG2* and *RAD14* are located on the right arm of chromosome XIII. Yeast.

[61] Rothstein R. (1991). Targeting, disruption, replacement, and allele rescue: Integrative DNA transformation in yeast. Methods Enzymol..

[62] Gietz R.D., Sugino A. (1988). New yeast-*Escherichia coli* shuttle vectors constructed with in vitro mutagenized yeast genes lacking six-base pair restriction sites. Gene.

[63] Rodicio R., Koch S., Schmitz H.P., Heinisch J.J. (2006). *KlRHO1* and *KlPKC1* are essential for cell integrity signalling in *Kluyveromyces lactis*. Microbiology.

[64] Schmitz H.P., Jendretzki A., Wittland J., Wiechert J., Heinisch J.J. (2015). Identification of Dck1 and Lmo1 as upstream regulators of the small GTPase Rho5 in *Saccharomyces cerevisiae*. Mol. Microbiol..

[65] Rippert D., Heppeler N., Albermann S., Schmitz H.P., Heinisch J.J. (2014). Regulation of cytokinesis in the milk yeast *Kluyveromyces lactis*. Biochim. Biophys. Acta.

