# Supplementary material for: A Versatile Toolset for Genetic Manipulation of the Wine Yeast Hanseniaspora uvarum
Source: Int J Mol Sci. 2023 Jan 17;24(3):1859. doi: 10.3390/ijms24031859 (PMC9915424; doi:10.3390/ijms24031859)
Supplement: Supplementary file 1 [file ijms-24-01859-s001.zip › ijms-2168178-supplementary.pdf]

Table S1. Oligonucleotides used in this work.

Number [1]	Designation [2]	Sequence (5′→ 3′) [3]
99.113	universal-47	CGCCAGGGTTTTCCCAGTCACGAC
14.111	reverse-90	GCTTCCGGCTCGTATGTTGTGTGG
06.252	AgTEFterm3out	TCGCCTCGACATCATCTGCCCAG
07.269	HuURA3forw	CCTTTCGGAGTTTAATAACCATATTCC
07.270	HuURA3rev	GATTCCATTTTTTGTGTGTGTGTG
09.183	HuLEU2forBam	gcgcggatccCTTGCTAGGTAAAGATGAAGCTG
09.184	HuLEU2revSal	gcgcgtcgacTAGAGTCCGCTCTCAAAGGGTG
09.185	HuADE2forKpn	gcgcggtaccGGGCCTTACTGGTGAATGCAGC
09.186	HuADE2revEco	gcgcgaattcGTTCTAAAACCCTGACTCCAC
10.225	AgTEFprom5out	CCTCAGTGGCAAATCCTAAC
20.341	HuURA3BamATG	gcgtggatccATGTCTACTCTAAGTTACGAAGCTAG
20.342	HuURA3revHind	ggcaagcTTATTCAGATACTCTTTTCAAATAAG
21.216	Hygro5out	GACAATTGCATCAAATCAGAAACAG
21.217	Hygro3out	GTTTTGGCTGATTCTGGTAATAGAAG
21.362	HuURA3 BglII	ctctagatCTTAAACACTATATAAACAAAGCTC
21.363	HuURA3 XhoI	cacgctcGAGTATAATCTCATAAAGTCCTTC
21.380	HuTEF1forEcoNot	gaggaattcgcggccgcCAAGTTTTACACGGTACCGTCAGAGAAATGA CAGCGGTaCGAAACATAGTCCATATCTCCG
21.381	HuTEF1revBam	gtgaggatccTTTTTTATAATTATTGTTAATGACTGG
22.111	HuARS3152Xhofor	ggtgctcgaGTTTTATTATTGGTTTTGAATGGTTAG
22.113	HuARS3152Bglrev2	ggcgagatctAACTTTTTCTTTGCCGAAAACAAAG
22.136	HuTEF1pforBgl	ggcgagatcTCAGAGAAATGACAGCGGTACG
22.137	HuTEF1prevSph	gcgtgcatgcTTTTTTATAATTATTGTTAATGACTGG
22.138	CreforSph	gcgtgcatgcTTAAATGTCCAATTTACTGACCG
22.139	CrerevEco	gcgtgaaTTCCTCGGTGGCATTCATCACGAAATG
22.140	Huade2del5loxP	AAAACATATTACAATAAAGTTTGGAAAAAACCCATAAAATAA CAATAGTAACAAGCTTCGTACGCTGCAGGTCGAC
22.141	Huade2del3loxP	ATAAAAAAAGAGGTAACATCACCTTTCAAGATTGACTACTTTG TTTGTATCTTGCATAGGCCACTAGTGGATCTG
22.142	Huura3del5loxP	AAGGACAAGAACCAAGGATTAAGACCCACCGATACATACCTC AATTGCAAAAGAGCTTCGTACGCTGCAGGTCGAC
22.143	Huura3del3loxP	ATTAAATATGTAGTCTACTTTAGGAAATAATATATATGTGTCGG GTAACACTTAGCATAGGCCACTAGTGGATCTG
22.144	Huhis3del5loxP	TTTGAATGATTACTACACTGAACTAAGAAACACCACCTAACAG ATCCGAAAAAGCTTCGTACGCTGCAGGTCGAC
22.145	Huhis3del3loxP	TATGTCTGTATTTTGCCTTTTTTTTTTATTATTAATATTGTCAAGA ATATCAAGCATAGGCCACTAGTGGATCTG
22.146	HuHIS3fornEco	GAAGTTAAAGAATTCAAATATG
22.147	HuHIS3revnBam	ATCTTAGAGGATCCAATCATTCCAAG
22.153	AgTEF2prevBam	gagaggatccGGTTGTTTATGTTCGGATGTGATG
22.154	YEp195HuSUI2p	TATATATACGCATATGTAGTGTTGAAGAAACATGAAATTGCCC AGTATTCTTGCTTGATGGATAACTTGAAGG
22.155	YEp195ARS1rev	AATAACTGATATAATTAAATTGAAGCTCTAATTTGTGAGTTTAG TATACATCTTTTTCTTTGCCGAAAACAAAG
22.156	HuARS1forXhowoHind	cactctcgAGCTTGGAGTAAAAGCAAACTCCC
22.158	3181HuARSrevBgl	ggtgagaTCTTTCACATGTAAACAAACTATGCGG
22.159	HuARSseqout	GTTATATTTGGTAAAATAAACGG
22.168	HuARS1for-100Xho	gtgactcgaGTTATATTTGGTAAAATAAACGG
22.169	HuARS1revHindBgl	CTAGTAGATCTAAgCTTTTTCTTTGCCGAAAACAAAG

22.240	HuADE2wvorNsi	GAAAATAATTCAACCAAATGCATG
22.241	HuADE2wnach	ATGGTATGTTTCTTAATCCAAGGG
22.244	Huleu2del5	ATAATGAAATGGAAATAACACCATACACTATATTAATAATATA TACAAATAAAAAGCTTCGTACGCTGCAGGTCGAC
22.245	Huleu2del3	TAAATGAACAAAAAAAATATGGCAAAGAGTTATTATTTAAGTT CTAGTATATAATGCATAGGCCACTAGTGGATCTG
22.246	HuLEU2wvor	GTTGTTATTATCAGTTCAGGTATGAG
22.247	HuLEU2wnach	GGTCATTAGAAAATACATGCTTTATG
22.249	HuHIS3wvorBam	gagaggatccGTTGGATCACGAATTGGG
22.250	HuHIS3wnachHind	GGCCTAAGCTTATATTTTCTTTCTACAATG
22.257	HuHIS3Hindfor	gcgcaagcTTCAAATATGTCACCATGGAAG
22.258	HuHIS3Salrev	gcgagtcgACTAATAAAGCTAAAGATTCGTG
22.259	HuURA3longflfor	GAGATGTTTAAGTAGAAAAAATTG
22.265	HuLEU2forNhe	CCTTGCTAGCTAAAGATGAAGCTGATAAACTAAC
22.266	HuURA3forNhe	gcgagctaGCCGTTGAAAAGTTGTTACAGG
22.267	HuURA3revSal	gcgagtcgacAGAGATGTTTAAGTAGAAAAAATTG
22.270	HuTEF1pseqin	GGTTTTTCTTAAACATTATGAAGTTCTTG
22.305	HuLEU2wwvor	GACGAATGTGTTGACTACCTAGTTG
22.306	HuLEU2wwnach	GGAGTTGCACAAAGATGGTGTTG

[1] numbers refer to the position of the oligonucleotide in our stock collection, please refer to those for requests

[2] names generally indicate for which gene this primer was used, a restriction site introduced, or the position relative to the gene in case of deletions, i.e. 5′ or 3′ to the open reading frame; "vor" and "nach" derive from German and indicate forward and reverse, respectively

[3] sequences printed in red and small letters indicate those not homologous to the template DNA, either introducing restriction sites for cloning, or introducing a mismatch; sequences in red print and capital letters in the larger oligonucleotides indicate the part annealing to the deletion cassettes; sequences in blue print correspond to naturally occuring restriction sites (or parts of them)
